# Pharmacokinetics and Safety of Multiple-Dose Alpelisib in Participants with Moderate or Severe Hepatic Impairment: A Phase 1, Open-Label, Parallel Group Study

**DOI:** 10.7150/jca.82736

**Published:** 2023-05-21

**Authors:** Thomas Marbury, Mona El-Hashimy, Lars Blumenstein, Franck Letellier, Tirtha Sengupta, Sebastien Lorenzo, Richard Alfred Preston

**Affiliations:** 1 Orlando Clinical Research Center, Orlando, USA.; 2 Novartis Pharmaceuticals Corporation, East Hanover, USA.; 3 Novartis Institutes for BioMedical Research, Basel, Switzerland.; 4 Novartis Pharma S.A.S, Paris, France.; 5 Novartis Healthcare Pvt. Ltd, Hyderabad, India.; 6 Novartis Pharma AG, Basel, Switzerland.; 7 Clinical Pharmacology Research Unit, Division of Clinical Pharmacology Department of Medicine, Miller School of Medicine, University of Miami, Miami, USA.; 8 Katz Family Drug Discovery Center, University of Miami, Miami, USA.

**Keywords:** Alpelisib, BYL719, Hepatic impairment, Pharmacokinetics

## Abstract

The pharmacokinetics (PK) and safety of single-dose alpelisib (300 mg) were assessed in participants with moderate to severe hepatic impairment (n = 6 each) compared with their matching healthy controls (n = 11). Blood samples were collected upto 144 hours post-dose and evaluated by liquid chromatography-tandem mass spectrometry (LC-MS/MS) assay. The primary PK parameters (maximum plasma concentration [C_max_], area under the curve [AUC]_inf_ and AUC_last_) and secondary PK parameters (AUC_0-t_, apparent total body clearance [CL/F], apparent volume of distribution [Vz/F], time of maximum observed concentration [T_max_], and half-life [T_1/2_]) of oral alpelisib 300 mg were determined from individual plasma concentration-time profiles using non‑compartmental analysis. C_max_ of alpelisib decreased by approximately 17% in the moderate hepatic impairment group vs. the healthy control group (geometric mean ratio; GMR [90% confidence interval; CI], 0.833 [0.530, 1.31]). C_max_ in the severe hepatic impairment group was comparable to that of the healthy control group (GMR [90% CI], 1.00 [0.636, 1.58]). AUC_last_ for alpelisib decreased by approximately 27% in the moderate hepatic impairment group vs. the healthy control group (GMR [90% CI], 0.726 [0.487, 1.08]). AUC_last_ was 26% higher in the severe hepatic impairment group compared with the healthy control group (GMR [90% CI], 1.26 [0.845, 1.87]). Overall, 3 participants (13.0%) experienced at least 1 adverse event which were either grade 1 or 2. Adverse events did not lead to study drug discontinuation. No grade 3 or 4 adverse events, serious adverse events or deaths were reported. The results indicate that a single dose of alpelisib was well tolerated in this study population. There was no significant impact of moderate or severe hepatic impairment on the exposure of alpelisib.

## Introduction

The phosphatidylinositol 3-kinase (PI3K) signaling pathway contributes to several processes that are critical in mediating many aspects of cellular function, including nutrient uptake, anabolic reactions, cell growth, and survival 1. The PI3K pathway is activated by multiple factors, including diverse oncogenic genomic alterations in *PIK3CA, PIK3R1, PTEN,* and other critical genes, which can serve as targets for anticancer therapy 1-3.

Alpelisib (BYL719; an oral, class I α-specific PI3K inhibitor belonging to the 2-aminothiazole class of compounds) strongly inhibits the PI3Kα isoform (both wildtype and mutant) over the β, δ, and γ isoforms, and is inactive against most other kinases 4. Antitumor activity of alpelisib has been demonstrated in preclinical and early phases of clinical studies as a single agent and in combination with endocrine therapy 5, 6. In the phase 3, SOLAR-1 study (NCT02437318), alpelisib plus fulvestrant significantly prolonged median progression-free survival versus placebo plus fulvestrant in patients with *PIK3CA*-mutated, hormone receptor-positive, human epidermal growth factor receptor 2-negative advanced breast cancer (11.0 vs 5.7 months; P<0.001) 7.

Alpelisib is currently being investigated in combination with other drugs in various oncology indications and is anticipated to be used in participants with co-existing morbidities, including hepatic impairment. Hepatic dysfunction results in pathophysiological changes that lead to variable and difficult-to-predict effects on drug pharmacokinetics (PK) 8. It is therefore important to determine the impact of hepatic impairment on the PK of alpelisib.

The first step in the metabolism of alpelisib *in vivo* is amide hydrolysis to BZG791, which is the major circulating metabolite in plasma. The metabolism of alpelisib to BZG791 is unlikely to be governed only by the liver, since it is primarily metabolized through both chemical and multi‑enzymatic amide hydrolysis, with only limited contribution from CYP3A4. As unchanged alpelisib can be eliminated by hepatobiliary export and/or intestinal secretion, liver metabolism and transport may account for an estimated fraction greater than 20% of the eliminated alpelisib 9. In order to allow for safe and efficacious use of alpelisib in participants with impaired liver function, the impact of liver impairment on the PK of alpelisib and its primary metabolite BZG791 (although pharmacologically inactive) needs to be characterized.

The likelihood of an effect of mild hepatic impairment on alpelisib PK may be very low because of the large contribution of non-hepatic metabolic pathways, biliary excretion, and active intestinal secretion. Therefore, the study was conducted in participants with moderate and severe hepatic impairment. The objective of this phase 1, open-label, multicenter, parallel-group study (NCT02624557) was to characterize the PK and safety profile of alpelisib in participants with moderate and severe hepatic impairment (by Child-Pugh classification) and to develop dosing recommendations based on the findings. Here, we report the results from the final analysis of this study.

## Materials and Methods

### Study Design

**Figure 1:**
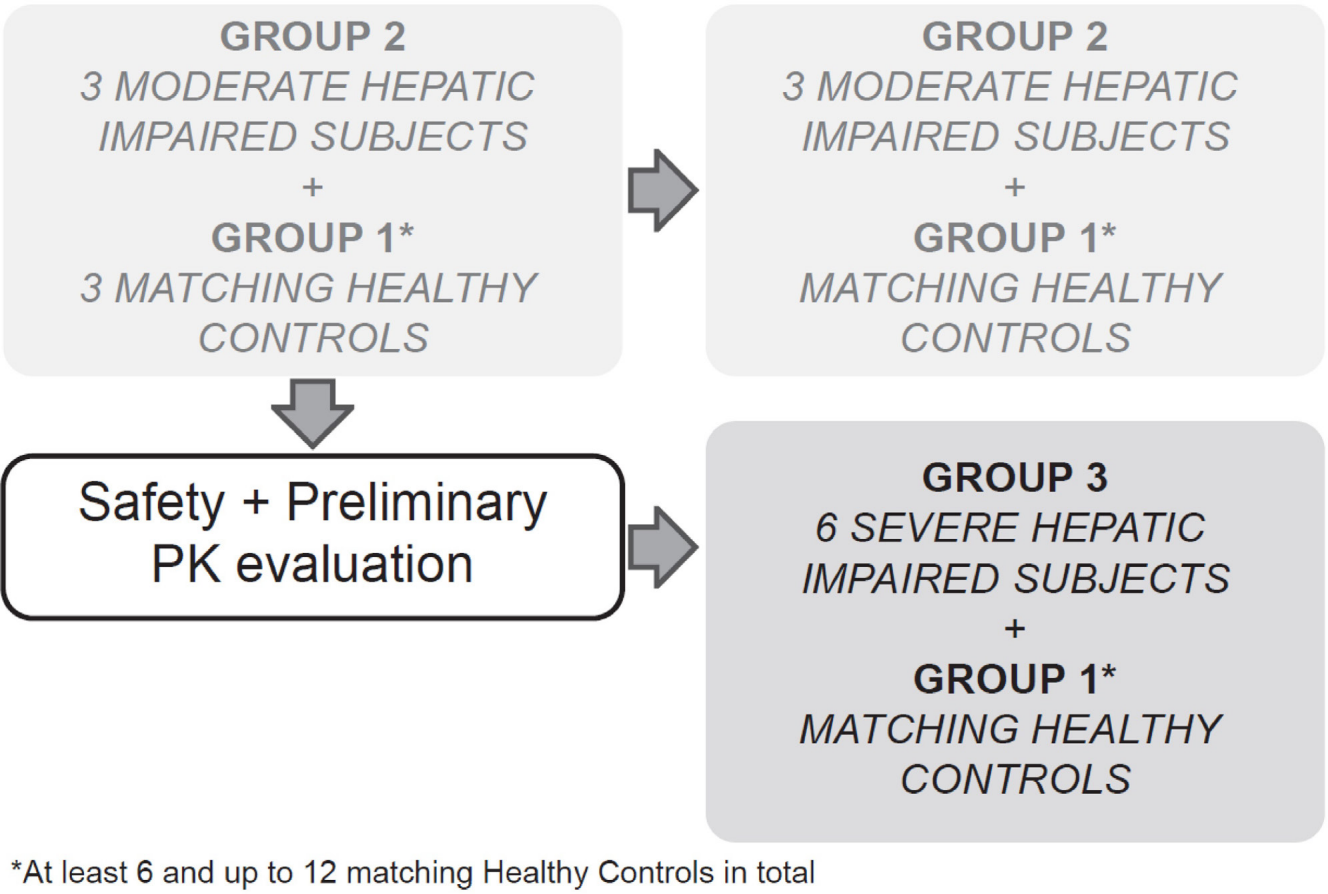
Study design

This multicenter, open-label, parallel-group study sequentially enrolled participants with moderate (Child-Pugh B or Group 2, score 7-9) and severe (Child-Pugh C or Group 3, score 10-15) hepatic impairment to assess the PK of alpelisib in participants with impaired hepatic function compared with healthy participants (Group 1, with apparent normal liver function) after a single dose of 300 mg alpelisib under fasted conditions (Figure 1). Enrollment commenced with three participants from the moderate hepatic impairment group. The enrollment of healthy matching controls did not start before his/her matching hepatic impaired participant had completed the end of study (EOS) visit. While enrollment in the moderate hepatic impairment group continued, an evaluation for safety and preliminary PK was made after the first 3 moderate impaired participants and their 3 matching healthy controls completed the study evaluations up to and including the EOS visit before starting enrollment into the severe hepatic impairment group.

The total study duration for each participant was approximately 7 weeks, which comprised a 21‑day screening period (Day -21 to Day -1), an 8-day confinement period (Day -1 to Day 7), and a follow-up safety period ending at least 30 days (+5 days) after alpelisib administration to follow up on ongoing adverse events (AEs), concomitant medication, and the occurrence of serious adverse events (SAEs).

This study was conducted in accordance with the Good Clinical Practice guidelines and the Declaration of Helsinki. An independent ethics committee and institutional review boards approved the study protocol and all amendments at each participating center. Written informed consent was obtained from all participants.

### Study Population

The study population consisted of participants (age, 18-75 years; weight, 50-120 kg; body mass index (BMI), 18.0-36.0 kg/m^2^) with moderate hepatic impairment (n=6) defined by Child-Pugh category B (7 to 9 total points) or severe hepatic impairment (n = 6) defined by Child-Pugh category C (10 to 15 total points; Table 1), who were otherwise healthy (exhibited physical signs consistent with stable hepatic impairment and were free of significant medical disorders unrelated to their hepatic disorder), and matched healthy control participants (matched by sex, race, age [±10 years], and body weight [±10%]; n = 12).

Key exclusion criteria included any surgical or medical condition or medical history that could affect the PK of alpelisib, use of any medication or food supplement 14 days prior to dosing or during the study that could affect the PK of alpelisib, medical history of liver transplant or immunosuppressant therapy, diabetes mellitus or fasting plasma glucose (FPG) levels > 160 mg/dL or > 8.8 mmol/L, donation or loss of ≥ 400 mL blood or plasma < 8 weeks prior to dosing, and history of psychiatric illness within the past 2 years. Detailed eligibility criteria can be found in Table S1.

### Study Assessments

#### Pharmacokinetic sample collection and analysis

Blood samples for assessing plasma concentration-time profiles of alpelisib were collected in K3-EDTA from all participants at pre-dose and at 0.5, 1, 1.5, 2, 2.5, 3, 4, 6, 8, 12, 24, 48, 72, 96, and 144 hours post-dose. Plasma samples were stored at -70°C.

Plasma concentrations of alpelisib and BZG791 were determined by a previously validated liquid chromatography-tandem mass spectrometry (LC-MS/MS) assay. The lower limits of quantitation (LLOQ) are currently 5.0 ng/mL for alpelisib and 1.0 ng/mL for BZG791. Values below the assay LLOQ were reported as 0 ng/mL.

#### Plasma protein binding sample collection and analysis

For plasma protein binding (PPB) analysis, blood samples were collected in K3-EDTA at two time points, pre-dose prior to alpelisib administration for the PPB of BZG791 and 3 hours after administration for the PPB of 14C-alpelisib. Plasma samples were stored at -20°C until analyzed.

Protein binding was determined by ultrafiltration method. Plasma, spiked with the intended compound (14C-alpelisib or BZG791), was introduced into ultrafiltration devices and the ultrafiltrate was obtained. Plasma protein binding was calculated based on compound concentration determined in the ultrafiltrate and in the spiked plasma sample before ultrafiltration. Stability was assessed in previous assay; though nonspecific adsorption was not investigated for this assay, neither analytes is known for unspecific binding from the in vitro assays performed with alpelisib or BZG791.

### Safety Assessments

The safety of single-dose oral alpelisib 300 mg was assessed throughout the study by recording AEs, clinical laboratory parameters, electrocardiograms (ECGs), and physical examinations; event severity (according to National Cancer Institute Common Terminology Criteria for Adverse Events [NCI‑CTCAE] version 4.03) and relationship to study drug were also recorded.

### Statistical Analysis

#### Population Size

The sample size (six participants per hepatic impairment group with a within-study control population) was based on practical considerations and guidance from the United States Food and Drug Administration and European Medicines Agency 10, 11.

#### Pharmacokinetic Analyses

The primary PK parameters (maximum concentration [C_max_], area under the curve from time zero to infinity [AUC_inf_] or AUC_last_) and secondary PK parameters (AUC_0-t_, apparent total body clearance [CL/F], apparent volume of distribution [Vz/F], time of maximum observed concentration [T_max_], and half‑life [T_1/2_]) of oral alpelisib 300 mg were determined from individual plasma concentration-time profiles using non-compartmental analysis (Phoenix WinNonlin Version 6.4 - Pharsight, Mountain View, CA).

Log-transformed parameters (C_max_, AUC_last_ and AUC_inf_) for both total and unbound alpelisib were analyzed by means of an analysis of covariance (ANCOVA) model, with hepatic function group as the fixed effect; supportive analyses were performed using the same model, with age, weight, sex, and race as covariates. The differences between the control group and each one of the hepatic function groups and the two-sided 90% confidence intervals (CIs) were derived from the model. These were back‑transformed to obtain the point estimates and the 90% CIs for the ratios of the geometric means on the original scale.

## Results

Of the 23 enrolled participants, 11 were in the healthy control group, six were in the moderate hepatic impairment group, and six were in the severe hepatic impairment group. All six participants in each impairment group were individually matched with a participant from the healthy control group. One participant from the healthy control group served as a matching control for two participants (one participant in the moderate hepatic impairment group and one participant in the severe hepatic impairment group). All participants completed the study and were included in the safety and PK analyses sets. Most participants were male (56.5%) and Caucasian (91.3%); age, gender, body weight, and other baseline characteristics were similar across treatment groups. Median creatinine clearance for all participants was 127.28 mL/min (normal); all participants had normal creatinine clearance except in one participant in Group 3 who had a mild renal impairment (56.81 mL/min) (Table 2).

### Pharmacokinetics

**Figure 2:**
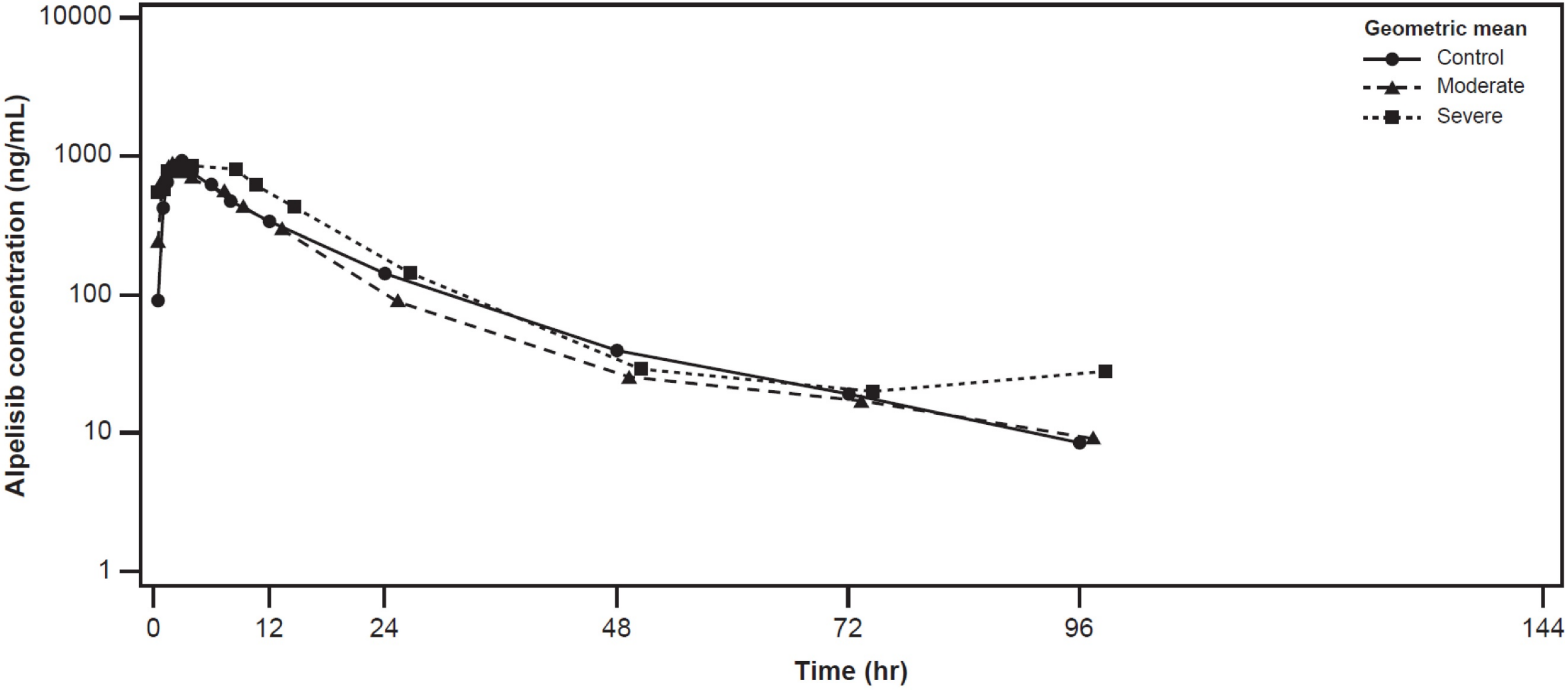
Geometric mean (SD) concentration-time profiles for plasma alpelisib by hepatic function group (Pharmacokinetic analysis set)

**Figure 3:**
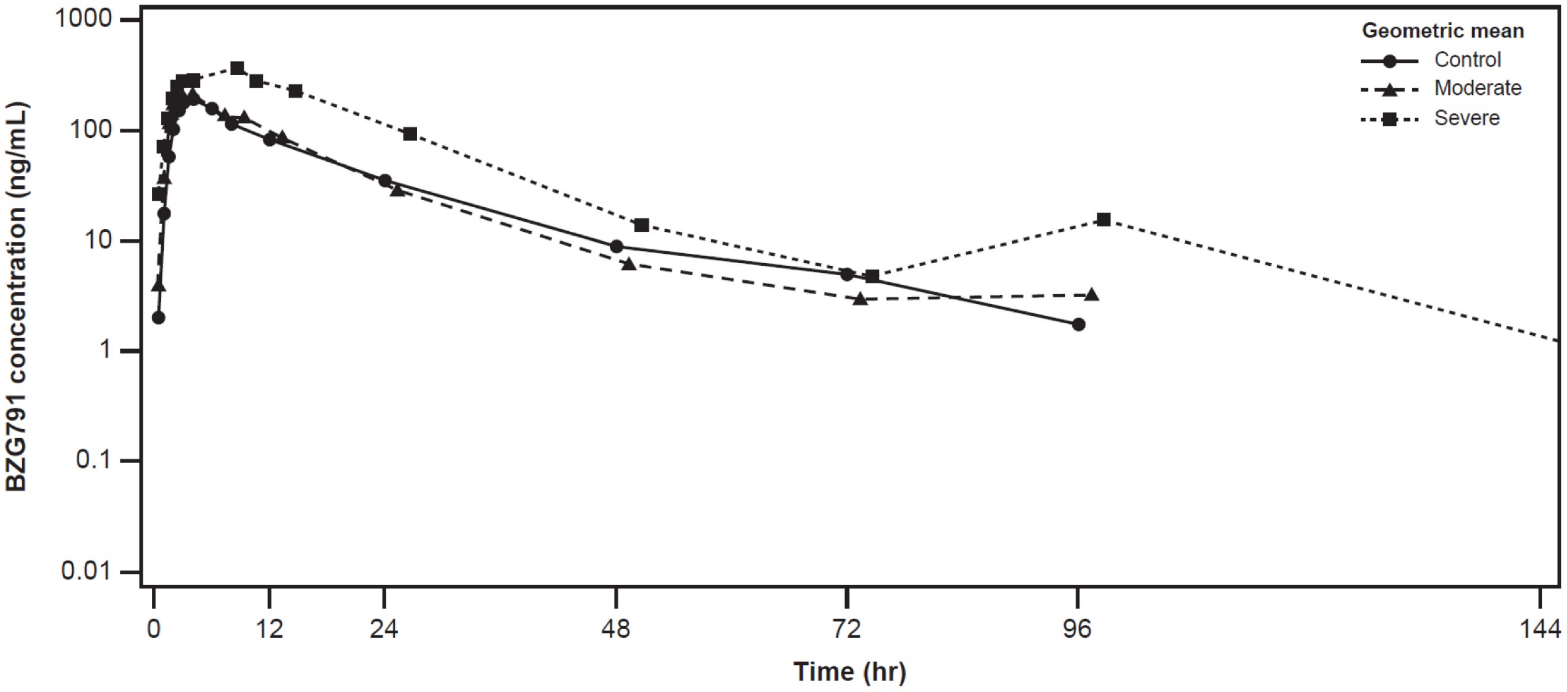
Geometric mean (SD) concentration-time profiles for plasma BZG791 by hepatic function group (Pharmacokinetic analysis set)

The plasma concentration-time profiles of alpelisib were largely similar across the healthy control, moderate hepatic impairment, and severe hepatic impairment groups (Figure 2). The plasma concentration-time profiles of BZG791 were largely similar in the healthy control and moderate hepatic impairment groups. Plasma concentrations were consistently higher in the severe hepatic impairment group from around 12 hours to 144 hours post-dose (Figure 3).

C_max_ of alpelisib decreased by approximately 17% in the moderate hepatic impairment compared with the healthy control group (geometric mean ratio; GMR [90% CI], 0.833 [0.530, 1.31]). C_max_ in the severe hepatic impairment group was comparable to that of the healthy control group (GMR [90% CI], 1.00 [0.636, 1.58]) (Table 3).

AUC_last_ for alpelisib decreased by approximately 27% in the moderate hepatic impairment group compared with the healthy control group (GMR [90% CI], 0.726 [0.487, 1.08]). AUC_last_ was 26% higher in the severe hepatic impairment group compared with the healthy control group (GMR [90% CI], 1.26 [0.845, 1.87]). Values of AUC_inf_ were similar to those of AUC_last_ across the hepatic impairment groups (Table 3).

C_max_ of BZG791 was comparable between the moderate hepatic impairment and healthy control groups (GMR [90% CI], 0.942 [0.639, 1.39]). In the severe hepatic impairment group, C_max_ increased by 74% (GMR [90% CI], 1.74 [1.18, 2.56]) (Table 4).

AUC_last_ for BZG791 decreased by approximately 12% in the moderate hepatic impairment group compared with the healthy control group (GMR [90% CI], 0.883 [0.598, 1.30]). It increased by 145% in the severe hepatic impairment group vs. the healthy control group (GMR [90% CI], 2.45 [1.65, 3.62]). Values of AUC_inf_ were similar to those of AUC_last_ across the hepatic impairment groups (Table 4). The metabolic ratio of BZG791 also increased (geometric mean [range], 0.472 [0.323 to 0.580]) in participants in the severe hepatic impairment group compared with participants in the healthy control and moderate hepatic impairment groups.

The average protein binding values of both alpelisib and BZG791 by group were also similar, indicating no significant effect of hepatic impairment on protein binding (Supplementary Table S2, Table S3, and Table S4). The PK parameter statistics for unbound alpelisib and BZG791 were similar to those of total alpelisib and total BZG791 in the healthy control and moderate hepatic impairment groups but were increased in the severe hepatic impairment group (the values of which also exhibited greater variability), which may be because of a slightly increased levels of fraction unbound in the severe hepatic impairment group.

### Safety

Of the 23 participants treated, three (13.0%) experienced at least one AE. All AEs were of either grade 1 (nausea and dizziness) or grade 2 (increased blood pressure) severity; there were no grade 3/4 AEs. No AEs were reported in the severe hepatic impairment group (Table 5). No clinically significant changes from baseline in vital signs or hematology and biochemistry laboratory values were reported in the study. No deaths, SAEs, or other significant AEs were reported in the study.

## Discussion

The objective of this study was to characterize the PK and safety profile of a single, oral 300 mg dose of alpelisib, administered in the fasted state, in participants with moderate and severe hepatic impairment (by Child-Pugh classification). The healthy control participants in this study were enrolled based on matched demographics with hepatic impaired participants with respect to age, race, sex, and body weight.

Compared with the healthy control group, C_max_ of alpelisib was around 17% lower in the moderate hepatic impairment group. Drug exposures as assessed by AUC_last_ and AUC_inf_ were both around 27% lower in the moderate hepatic impaired group compared with the healthy control group. C_max_ of alpelisib in the severe hepatic impairment group was comparable to that of the healthy control group. Drug exposure as assessed by AUC_inf_ provided similar results as drug exposure assessed by AUC_last_.

It is likely that the differences in alpelisib maximum concentration and exposure across the hepatic impairment groups are an effect of variability rather than a true effect of hepatic impairment, especially with the effect showing an opposite trend in the moderate hepatic impairment group compared with the severe hepatic impairment group. Some variability is generally expected with small sample sizes (six participants in each of the hepatic impairment groups), and a prior food effect study with alpelisib had shown that alpelisib PK variability is higher in the fasted state compared with the fed state.

No apparent relationship was found between any PK parameter of alpelisib and a hepatic laboratory parameter.

The PK parameters of the metabolite BZG791, C_max_, AUC_last_, and AUC_inf_, were comparable in the moderate hepatic impairment group and the healthy control group, but were significantly increased (C_max_ by 74%, AUC_last_ by 145%, and AUC_inf_ by 139%) in the severe hepatic impairment group because of higher exposure in the severe hepatic impairment group compared with the healthy control group. The metabolic ratio of BZG791 was also significantly increased in the severe hepatic impairment group compared with the healthy control group.

The current disposition model of alpelisib based on the results of the human ADME study postulates that approximately 73% of alpelisib is hydrolyzed to BZG791 systemically (i.e., extrahepatically), with 22% contribution of CYP3A4-mediated metabolism and a minor (4%) contribution of renal excretion. While alpelisib and BZG791 are substrates for BCRP, export of BCRP is not expected a large role in the disposition of alpelisib (and thus sensitive to any polymorphisms related to transporter expression) but may play a role in the disposition of BZG791 9.

Hepatic impairment should result in a loss or reduction of metabolic activity and transporter activity in the liver. BZG791 exposure was also changed in the mild to moderate impairment group, suggesting that the hydrolysis pathway outside of the liver is not affected by the loss of oxidative metabolism or export in the liver, which is supportive of the current model. In severe hepatic impaired participants, the exposure of BZG791 was found to be significantly increased. This observed increase in BZG791 PK parameters and metabolic ratio without the exposure of alpelisib being significantly impacted in the severe impairment group compared with the control group may be explained either by an inhibition of BZG791 disposition pathways (e.g. via BCRP as it is not considered to be metabolized further) or by a compensatory mechanism. A confounding impact on the results by genetic polymorphisms of BCRP or concurrent impaired renal function (observed in one participant in the severe hepatic impairment group) can be considered unlikely due to the low contribution to the disposition of alpelisib itself.

Protein binding values in the healthy control, moderate hepatic impairment, and severe hepatic impairment groups were similar for both alpelisib and BZG791 and was in line with previously reported *in vitro* protein binding values. The GMRs of unbound alpelisib and unbound BZG791 were similar to that of total alpelisib and total BZG791, respectively. However, the values of the PK variability for unbound alpelisib and BZG791 were found to be increased across all treatment groups compared with that of total bound alpelisib and BZG791.

Collectively, these results indicate that moderate or severe hepatic impairment has no impact on the clearance, elimination, or distribution of alpelisib. Therefore, it is reasonable to conclude that mild hepatic impairment also has no impact. The increase in BZG791 exposure in the severe impairment group is, however, indicative of a shift in the metabolic pathways. The data from this study has led to the alpelisib label recommendations for patients with hepatic impairment.

Very few AEs were reported in this study, mainly of grade 1 or grade 2 severity. No clinically significant changes from baseline in vital signs or hematology and biochemistry laboratory values were reported in the study. No deaths, SAEs, or other significant AEs were reported in the study.

A single dose of alpelisib was well tolerated in this study population. No significant impact of moderate or severe hepatic impairment was observed on the clearance, elimination, or distribution of alpelisib. In conclusion, this analysis supports that no dose adjustment of alpelisib is required in participants with mild, moderate, or severe hepatic impairment.

## Supplementary Material

Supplementary tables.

## Figures and Tables

**Table 1:** Child-Pugh classification and liver parameters at screening by hepatic function group (Full analysis set)

Hepatic impairment classification	Moderate(total score 7-9; n = 6), n (%)	Severe(total score 10-15; n = 6), n (%)
**Encephalopathy**		
Grade 1**-**2	6 (100)	6 (100)
**Ascites**		
Slight	6 (100)	0
Moderate	0	6 (100)
**Total bilirubin (mg/dL)**		
<2	6 (100)	0
2-3	0	2 (33.3)
>3	0	4 (66.7)
**Serum albumin (g/dL)**		
>3.5	6 (100)	1 (16.7)
2.8-3.5	0	3 (50.0)
<2.8	0	2 (33.3)
**International normalized ratio**		
<1.7	5 (83.3)	3 (50.0)
**Prothrombin time (seconds over control)**		
< 4	1 (16.7)	2 (33.3)
4 to 6	0	1 (16.7)
**Score**		
7	6 (100)	0
10	0	3 (50.0)
11	0	1 (16.7)
12	0	1 (16.7)
13	0	1 (16.7)

Moderate = Child-Pugh class B; Severe = Child-Pugh class C.

**Table 2:** Participant demographics by hepatic function group

Characteristic	Control (N = 11)	Moderate (N = 6)	Severe (N = 6)	All Participants (N = 23)
Age, median (range), years	57.0 (47.0 - 68.0)	57.0 (53.0 - 62.0)	62.5 (48.0 - 66.0)	58.0 (47.0 - 68.0)
Sex, n (%)				
Male	6 (54.5)	3 (50.0)	4 (66.7)	13 (56.5)
Female	5 (45.5)	3 (50.0)	2 (33.3)	10 (43.5)
Race, n (%)				
Caucasian	10 (90.9)	5 (83.3)	6 (100)	21 (91.3)
Black	1 (9.1)	1 (16.7)	0	2 (8.7)
Ethnicity, n (%)				
Hispanic/Latino	5 (45.5)	4 (66.7)	3 (50.0)	12 (52.2)
Mixed Ethnicity	2 (18.2)	0	0	2 (8.7)
Other	4 (36.4)	2 (33.3)	3 (50.0)	9 (39.1)
Weight, median (range), Kgs	82.60 (55.1 - 112.9)	74.70 (52 - 103.1)	82.75 (68.7 - 91.5)	81.50 (52 - 112.9)
Height, median (range), cms	166.5 (150 - 180)	164.1 (159 - 174)	169.5 (158 - 185)	166.3 (150 - 185)
Body mass index, median (range), Kg/m^2^	28.14 (24.24 - 34.85)	28.295 (19.33 - 36.17)	28.43 (21.99 - 33.8)	28.14 (19.33 - 36.17)
Creatinine clearance, median (range), mL/min	112.29 (81.03 - 201.61)	131.16 (111.27 - 140.97)	137.725 (56.81 - 231.53)	127.28 (56.81 - 231.53)

**Table 3:** Summary of statistical analysis of primary PK parameters for alpelisib by hepatic function group (Pharmacokinetic analysis set)

PK parameter (unit)	Group (Control, N=11; Moderate, N=6; Severe, N=6)	Adjusted geo-mean	Comparison(s)	Group comparison		
				Geo-mean ratio	90% CI	
					Lower	Upper
Cmax (ng/mL)	Control	1180	-	-	-	-
	Moderate	986	Moderate/Control	0.833	0.530	1.31
	Severe	1190	Severe/Control	1.00	0.636	1.58
AUClast (ng*hr/mL)	Control	13300	-	-	-	-
	Moderate	9630	Moderate/Control	0.726	0.487	1.08
	Severe	16700	Severe/Control	1.26	0.845	1.87
AUCinf (ng*hr/mL)	Control	13700	-	-	-	-
	Moderate	9990	Moderate/Control	0.730	0.499	1.07
	Severe	17100	Severe/Control	1.25	0.859	1.83
Tmax (hr)	Control	2.02	-	-	-	-
	Moderate	1.75	Moderate/Control	-0.267	-	-
	Severe	2.75	Severe/Control	0.733	-	-

AUC_inf_, area under the curve from time zero to infinity; CI, confidence interval; C_max_, maximum blood concentration; T_max_, time at which C_max_ is reached. For T_max_, median is presented under 'Adjusted geo-mean', difference of medians under 'Geo-mean ratio'. Geometric mean ratio of the healthy control participant group and each one of the hepatic function groups was estimated using an ANCOVA model of the log-transformed PK parameters. Included in the model were hepatic function group as a fixed effect and sex as covariate for Cmax, hepatic function group, weight and sex as covariates for AUClast and AUCinf. The results were back transformed to get adjusted geometric mean, geometric mean ratio, and 90% CI.

**Table 4:** Summary of statistical analysis of primary PK parameters for BZG791 by hepatic function group (Pharmacokinetic analysis set)

PK parameter (unit)	Group (Control, N=11; Moderate, N=6; Severe, N=6)	Adjusted geo-mean	Comparison(s)	Group comparison		
				Geo-mean ratio	90% CI	
					Lower	Upper
Cmax (ng/mL)	Control	242	-	-	-	-
	Moderate	228	Moderate/Control	0.942	0.639	1.39
	Severe	421	Severe/Control	1.74	1.18	2.56
AUClast (ng*hr/mL)	Control	3110	-	-	-	-
	Moderate	2750	Moderate/Control	0.883	0.598	1.30
	Severe	7620	Severe/Control	2.45	1.65	3.62
AUCinf (ng*hr/mL)	Control	3200	-	-	-	-
	Moderate	2840	Moderate/Control	0.887	0.608	1.29
	Severe	7640	Severe/Control	2.39	1.64	3.49
Tmax (hr)	Control	4.00	-	-	-	-
	Moderate	4.00	Moderate/Control	0	-	-
	Severe	6.00	Severe/Control	2.00	-	-

AUC_inf_, area under the curve from time zero to infinity; CI, confidence interval; C_max_, maximum blood concentration; T_max_, time at which C_max_ is reached. For T_max_, median is presented under 'Adjusted geo-mean', difference of medians under 'Geo-mean ratio'. Geometric mean ratio of the healthy control participant group and each one of the hepatic function groups was estimated using an ANCOVA model of the log-transformed PK parameters. Included in the model were hepatic function group as a fixed effect and sex as covariate for Cmax, hepatic function group, weight and sex as covariates for AUClast and AUCinf. The results were back transformed to get adjusted geometric mean, geometric mean ratio, and 90% CI.

**Table 5:** Adverse events, regardless of study drug relationship, by hepatic function group, preferred term, and maximum grade (Safety set)

Primary system organ class Preferred term, n (%)^a^	Control (N = 11)	Moderate (N=6)	Severe (N=6)	All Participants (N=23)
Any Grade	1 (9.1)	2 (33.3)	0	3 (13.0)
Grade 1	1 (9.1)	1 (16.7)	0	2 (8.7)
Grade 2	0	1 (16.7)	0	1 (4.3)
Nausea	0	2 (33.3)	0	2 (8.7)
Blood pressure increased	0	1 (16.7)	0	1 (4.3)
Dizziness	1 (9.1)	0	0	1 (4.3)

^a^A participant with multiple occurrences of an adverse event under a hepatic function group is counted only once in the adverse event category for that group. A participant with multiple adverse events within a primary system organ class is counted only once in the total row at maximum severity grade.

## Abbreviations

ADME: absorption, distribution, metabolism and excretion
AE: adverse event
ANCOVA: analysis of covariance
AUC: area under the curve
AUC_0-t_: area under the concentration-time curve from time zero to time t
AUC_inf_: area under the concentration-time curve (AUC) from time zero to infinity
AUC_last_: area under the concentration-time curve (AUC) from time zero to the last measurable concentration sampling time (T_last_)
BCRP: breast cancer resistance protein
BMI: body mass index
Bpm: beats per minute
CI: confidence interval
CL/F: total body clearance of drug from the plasma
C_max_: maximum plasma concentration
CTCAE: Common Terminology Criteria for Adverse Events
ECG: electrocardiogram
EDTA: ethylene diamine tetraacetic acid
EOS: end of study
FPG: fasting plasma glucose
GMR: geometric mean ratio
LLOQ: lower limits of quantitation
LCMS/MS: liquid chromatography-tandem mass spectrometry
NCI: National Cancer Institute
PI3K: phosphatidylinositol 3'-kinase
PK: pharmacokinetics
PPB: plasma protein binding
SAE: serious adverse event
SD: standard deviation
T_max_: time of maximum observed concentration
T_1/2_: half-life
ULN: upper limit of normal
Vz/F: volume of distribution during terminal elimination phase
