# Supplementary material for: Pharmacokinetics and Safety of Multiple-Dose Alpelisib in Participants with Moderate or Severe Hepatic Impairment: A Phase 1, Open-Label, Parallel Group Study
Source: J Cancer. 2023 May 21;14(9):1571–8. doi: 10.7150/jca.82736 (PMC10266244; doi:10.7150/jca.82736)
Supplement: Supplementary file 1 — Supplementary tables. [file jcav14p1571s1.pdf]

Supplementary Tables

Table S1: Eligibility criteria for participant inclusion

Table S2: Summary of statistical analysis of unbound plasma PK parameters for alpelisib by hepatic function group (Pharmacokinetic analysis set)

Table S3: Summary of statistical analysis of unbound plasma PK parameters for BZG791 by hepatic function group (Pharmacokinetic analysis set)

Table S4: Summary of clinical plasma protein binding of ^{14}C-alpelisib and BZG791 (Pharmacokinetic analysis set)

Table S1: Eligibility criteria for participant inclusion

**Inclusion criteria**	
**Main inclusion criteria for all participants**	• Participants must provide written informed consent.<br>• Women must be of non-childbearing potential.<br>• Sexually active male participants must use a condom during intercourse while taking drug and for 1 week after stopping study medication and should not father a child in this period.<br>• Participants must weigh ≥50 kg and ≤120 kg and have a BMI of 18.0 to 36.0 kg/m^2.
**Additional inclusion criteria for matched control participants**	• Participants in good health as determined by no clinically significant findings from medical history, physical examination, vital signs, and ECG.<br>• Laboratory values (if not otherwise specified) within the reference range at the local laboratory, unless deemed not clinically significant by the investigator or designee.<br>• At screening and baseline, vital signs* are within the following ranges:<br>○ Body temperature: ≥35.0 and ≤37.5°C<br>○ Systolic blood pressure: ≥90 and ≤140 mm Hg<br>○ Diastolic blood pressure: ≥50 and ≤90 mm Hg<br>○ Pulse rate: ≥50 and ≤90 beats per minute
**The additional inclusion criteria for participants with hepatic impairment**	• Participants must have a score clinically determined and calculated as per the Child-Pugh classification and consistent with the degree of hepatic impairment in which study is currently enrolling.<br>• Stable Child-Pugh status within 28 days prior to dosing.<br>• Participants with stable hepatic impairment with other stable medical disorders such as hypertension, hyperlipidemia, hypothyroidism etc., are eligible, as long as they are considered appropriate for enrollment as determined by past medical history, physical examination, vital signs, ECG, and laboratory tests at screening and baseline.<br>• Absence of moderate to severe impaired renal function as indicated by any or all of the following criteria:<br>○ Creatinine clearance ≥45 mL/min as calculated using Cockcroft-Gault formula<br>○ Serum creatinine ≤1.5 × ULN<br>• Fasting serum lipase ≤3 × ULN<br>• At screening and baseline, vital signs* are within the following ranges:<br>○ Body temperature: ≥35.0 and ≤37.5°C<br>○ Systolic blood pressure: ≥90 and ≤150 mm Hg<br>○ Diastolic blood pressure: ≥50 and ≤100 mm Hg<br>○ Pulse rate: ≥50 and ≤100 beats per minute
**Exclusion criteria**	
**Main exclusion criteria for all participants**	• Women of childbearing potential.<br>• Fertile male participants unless the study participant and his female partner agree to comply with highly effective contraception for the duration of the study and up to 1 week following study drug administration and should not father a child in this period.<br>• Participant has received a liver transplant at any time in the past and is on immunosuppressant therapy.<br>• Any condition which, in the opinion of the investigator, is likely to interfere with the study conduct, such as uncontrolled infection, uncontrolled blood pressure variation (hypo- or hypertension), active gastrointestinal bleeding, or hospitalization within 14 days prior to dosing.

	• Smokers not willing to limit the use of tobacco or products containing nicotine to approximately 10 cigarettes per day (or equivalent) starting 1 week prior to dosing until the last day of confinement in study center.<br>• Participant has had any surgical or medical condition which might significantly alter the absorption, distribution, metabolism, or excretion of drugs, or which may jeopardize the participant's safety in case of participation in the study. History of acute pancreatitis within 1 year of study entry.<br>• Participants with fasting plasma glucose levels >160 mg/dL or >8.88 mmol/L.<br>• Participants with medical history judged by the investigator as clinically significant for the risk of developing diabetes mellitus during the study.<br>• Dosing in any clinical investigation within 30 days of dosing or five half-lives of the investigational product, whichever is longer, or longer if required by local regulations.<br>• Contraindication or hypersensitivity to any drug or metabolites from similar class as study drug or to any excipients of alpelisib drug formulation<br>• Participant has used any herbal medications/supplements within 14 days prior to dosing. Herbal preparations are not allowed throughout the study.
**Additional exclusion criteria for matched control participants**	• Use of any prescription or non-prescription medication, dietary supplements, or vitamins during 14 days prior to dosing.<br>• A positive hepatitis B surface antigen or hepatitis C test result.<br>• History of drug or alcohol abuse within 1 month prior to dosing or evidence of such abuse as indicated by laboratory values at screening or baseline.<br>• Screening or baseline ECG: QTcF >450 msec for males; QTcF >460 msec for females; PR >200 msec; QRS complex >110 msec, or other morphological changes other than repolarization, nonspecific S-T or T-wave changes.
**The additional exclusion criteria for participants with hepatic impairment**	• Use of any prescription or non-prescription medication, dietary supplements, or vitamins that has the potential to interact with alpelisib, within 14 days prior to dosing or during the study. Concomitant medications without potential to interact with alpelisib must be stable in dose and dosing regimen within 14 days prior to dosing (diuretics given to treat ascites must be stable in dose and dosing regimen within 28 days) and must be discussed with and approved by the sponsor prior to participant enrollment.<br>• Participants requiring tapping (more frequently then every three months) for the management of ascites are excluded.<br>• Encephalopathy grade 3 or worse within 28 days prior to dosing.<br>• International normalized ratio >2.5 indicating impact of liver disease on coagulation.<br>• Total bilirubin >6 mg/dL.<br>• Corrected serum calcium <7.0 mg/dL.<br>• History of drug or alcohol abuse within 1 month prior to dosing or evidence of such abuse as indicated by laboratory values at screening or baseline. However, participants may participate in the trial if the positive drug screen is due to prescription drug use for a specific symptom such as insomnia or pain.<br>• Screening or baseline ECG: QTcF >480 msec for both genders.

*Measured after 5 minutes resting in sitting position
BMI, body mass index; ECG, electrocardiogram; QTcF, QT interval corrected using Fridericia's formula; ULN, upper limit of normal

Table S2: Summary of statistical analysis of unbound plasma PK parameters for alpelisib by hepatic function group (Pharmacokinetic analysis set)

PK parameter (unit)	Group (Control, N=11; Moderate, N=6; Severe, N=6)	Adjusted geo-mean	Comparison(s)	Group comparison		
				Geo-mean ratio	90% CI	
					Lower	Upper
Cmax (ng/mL)	Control	112	-	-	-	-
	Moderate	95.7	Moderate/Control	0.854	0.544	1.34
	Severe	153	Severe/Control	1.36	0.866	2.15
AUClast (ng*hr/mL)	Control	1250	-	-	-	-
	Moderate	1000	Moderate/Control	0.804	0.516	1.25
	Severe	2160	Severe/Control	1.73	1.11	2.70
AUCinf (ng*hr/mL)	Control	1290	-	-	-	-
	Moderate	1040	Moderate/Control	0.807	0.528	1.23
	Severe	2220	Severe/Control	1.72	1.12	2.64

AUC_{inf}, area under the curve from time zero to infinity; CI, confidence interval; C_{max}, maximum blood concentration.

Table S3: Summary of statistical analysis of unbound plasma PK parameters for BZG791 by hepatic function group (Pharmacokinetic analysis set)

PK parameter (unit)	Group (Control, N=11; Moderate, N=6; Severe, N=6)	Adjusted geo-mean	Comparison(s)	Group comparison		
				Geo-mean ratio	90% CI	
					Lower	Upper
Cmax (ng/mL)	Control	10.3	-	-	-	-
	Moderate	9.26	Moderate/Control	0.902	0.617	1.32
	Severe	26.4	Severe/Control	2.57	1.76	3.77
AUClast (ng*hr/mL)	Control	132	-	-	-	-
	Moderate	112	Moderate/Control	0.846	0.574	1.25
	Severe	478	Severe/Control	3.63	2.46	5.35
AUCinf (ng*hr/mL)	Control	135	-	-	-	-
	Moderate	115	Moderate/Control	0.849	0.584	1.24
	Severe	480	Severe/Control	3.54	2.43	5.16

AUC_{inf}, area under the curve from time zero to infinity; CI, confidence interval; C_{max}, maximum blood concentration.

Table S4: Summary of clinical plasma protein binding of ^{14}C-alpelisib and BZG791 (Pharmacokinetic analysis set)

Group	^{14}C-alpelisib, fu ± SD (%)	BZG791, fu ± SD (%)
Control	9.45 ± 0.691	4.24 ± 0.438
Moderate	9.72 ± 0.428	4.08 ± 0.451
Severe	12.8 ± 1.75	6.30 ± 1.56

Fu, The fraction unbound in plasma; SD, standard deviation.
